# 

**Published:** 1899-05-13

**Authors:** 


**Notices of Births, Deaths, and Marriages** are inserted in this column at a charge of 2s. 6d. for an announcement not exceeding four lines.

**MARRIAGE.**

On April 24th, at Holy Trinity Church, Eastbourne, by the Rev. H. G. Jameson, Vicar of St. Peter's, Eastbourne, John Perch Goodridge, late Sessions Judge of Jubbulpore, C.P. India, to Maria Louisa, third daughter of John Weston, of Anerley, and formerly of Brockley, S.E. (1,176)

### NOTICE TO CORRESPONDENTS.

All MS., Letters, Books for Review, and other matters intended for the Editor should be addressed The Editor, "The Hospital," The Hospital Building, 28 & 29, Southampton Street, Strand, W.C.

The Editor cannot undertake to return rejected MS., even when accompanied by stamped directed envelope.

**Notice to Advertisers and Subscribers.**

All Advertisements, Orders for Copies of The Hospital, and Business Communications should be addressed to **THE MANAGER (Not to the Editor),** The Hospital, The Hospital Building, 28 & 29, Southampton Street, Strand, London, W.C.

The Cost of Subscription, post free, is as follows:—Per week, 2½d.; per quarter, 2s. 8d.; per half-year, 5s. 3d.; per annum, 10s. 6d. Foreign:—Per annum, 15s. 2d., payable, in all cases, in advance.

**NOTICE.—Vol. XXV. of "The Hospital" (October, 1898, to March, 1899), in handsome cloth binding, is now on sale at the Office of this Journal, price 7s. 6d. Cases for binding the Numbers contained in this Volume 1s. 6d. Index to Vol. XXV., 2½d., post free.**

**The Monthly Part for May is now ready. Price 6d., by post 8d.**

### SCRAPS AND GLEANINGS.

The convalescent home in connection with the Bristol Royal Infirmary, in which the committee are to have 30 beds at their disposal, will be opened shortly.

Plans have been decided upon (subject to the Local Government Board's acceptance) for the erection of additional accommodation in connection with the infirmary at Romsey, which is required by the Local Government Board. It is proposed to erect a duplicate of the present building, and to use the two buildings for men and women respectively. The cost of this addition would be about £2,000.

The gift by the Primate of £1,000 out of the Marriott bequest, to the new wing of the Victoria Hospital, Folkestone, leaves only about £500 to be provided, which it is hoped will be speedily raised, and thus enable the new wing to be opened free from debt. The cost of this new wing is estimated at about £4,500, of which about £3,000 has been contributed to the Jubilee Fund by the public.

It is proposed to build a small wing to the Carmarthenshire Infirmary, at a cost of about £500, for the provision of additional sanitary offices.

The proposal to enlarge and improve the Norwich Isolation Hospital at an estimated cost of £11,380 has been definitely accepted. By this extension forty-two beds will be added, which will be contained in two new pavilions, viz., eighteen beds for typhoid and twenty-four for scarlet fever cases. In this way one of the existing pavilions will be set free and will be utilised for diphtheria cases.

Their Royal Highnesses the Duke and Duchess of York have graciously consented to open the new wing of the Boys' Home, of the National Refuges for Homeless and Destitute Children, Fortescue House, Twickenham, on Thursday, June 8th. This home, which accommodates 120 boys, was opened 20 years ago, on the work being transferred from the famous refuge in Queen Street, Holborn.

**Administrative Appointment.**

WOLVERHAMPTON AND STAFFORDSHIRE GENERAL HOSPITAL.

A Gentleman is REQUIRED to fill the OFFICE of HOUSE GOVERNOR and SECRETARY in this Institution. He must be unmarried, or a widower without the care of a family, be competent to manage the affairs of a large public charity, and must possess a thorough knowledge of accounts.

The remuneration will be £170 a year, with Board and Washing and Residence in the Institution. Security will be required to the amount of £500.

A collector and a junior clerk are kept.

Applications and testimonials, sealed and addressed to the Chairman of the Board of Management, must be received at the Hospital not later than Ten o'clock on FRIDAY MORNING, the 26th inst.

By order,
EDWIN A. WHITE,
(1,238) House Governor and Secretary.

Demy 8vo., with Portrait, cloth, extra gilt, 16s.

**GEORGE HARLEY, F.R.S.**

**The Life of a London Physician.**

Edited by his Daughter, Mrs. ALEC TWEEDIE.

"Does a reader thirst for adventure? Let him study Dr. Harley's experience at the time of the Crimean War; his escape from prison was little short of miraculous. In the matter of miscellaneous reminiscences Mrs. Alec Tweedie's book is a fruitful field."—*Liverpool Post.*

London: The Scientific Press, Ltd., 28 & 29, Southampton Street, Strand, W.C.

